# Spectrum of Pancytopenia in Adults Attending a Clinical Hematology Department: A Four-Year Experience From a Tertiary Care Center of Western India

**DOI:** 10.7759/cureus.24933

**Published:** 2022-05-12

**Authors:** Govind R Patel, Gopal R Prajapati

**Affiliations:** 1 Clinical Hematology, Dr. Sampurnanand Medical College, Jodhpur, IND; 2 Emergency Medicine, Dr. Sampurnanand Medical College, Jodhpur, IND

**Keywords:** aplastic anemia, megaloblastic anemia, acute leukemia, pancytopenia, etiology, clinico-hematological profile

## Abstract

Background

Pancytopenia is a common hematological condition encountered in clinical practice. Because there is a wide variation in causes of pancytopenia even in different populations of the same geographical region, identifying the pattern of underlying etiologies is crucial for proper management. This study was conducted to evaluate the clinico-hematological profile and different etiologies of pancytopenia among adults presenting to a clinical hematology department at a tertiary care hospital of Western India.

Methodology

This observational study was conducted over a period of four years on 546 adult patients aged 18-93 years presenting with pancytopenia. After obtaining a detailed clinical history and physical examination, all participants were subjected to relevant investigations including bone marrow examination.

Results

A slight male preponderance was observed, and the most common age group (24%) was 21-30 years. Pallor was the most common clinical feature, followed by generalized weakness and fever. The most common cause of pancytopenia was acute leukemia (17.9%), followed by megaloblastic anemia (15.4%), aplastic anemia (11.0%), hypersplenism (7.8%), multiple myeloma (6.6%), and myelodysplastic syndrome (5.3%).

Conclusions

Acute leukemia was observed to be the most common cause of pancytopenia in this study, which is in contrast to various other studies from India conducted in different departments. Identification of etiologies of pancytopenia among patients attending hematology departments in various regions is expected to be useful in formulating diagnostic algorithms and management strategies, which can help clinicians to better manage such patients.

## Introduction

Pancytopenia refers to a simultaneous decrease in erythrocytes, leukocytes, and platelets resulting in anemia, leukopenia, and thrombocytopenia. It is a fairly common hematological condition encountered in routine clinical practice. Clinical features of pancytopenia usually result from bone marrow (BM) failure such as pallor, generalized weakness, dyspnea, bleeding manifestations, prolonged fever, and increased tendency to infections [1].

Pancytopenia is a clinical manifestation of several underlying disorders including non-malignant and malignant conditions. Various mechanisms of the development of pancytopenia have been proposed. Major factors include reduction in hematopoietic cell production as in aplastic anemia, infiltration of BM by abnormal cells as in hematological malignancies, ineffective hematopoiesis with cell destruction as in megaloblastic anemia, antibody-mediated destruction of cells as in autoimmune disorders, and splenic sequestration of cells as in hypersplenism [2]. The cause and severity of the underlying disorder guide the treatment and prognosis of patients with pancytopenia [3]. Early diagnosis and specific treatment cure the majority of cases of pancytopenia. In other cases, early diagnosis and timely initiation of supportive treatment reduce morbidity and mortality, thereby improving the quality of life [4].

The etiology of pancytopenia varies in different populations depending on numerous factors, such as variations in age, gender, nutrition, geographical distribution, living standard, exposure to cytotoxic drugs or toxins, exposure to infections, and genetic and mutation profile [5]. Variation in the etiology of pancytopenia is observed in different countries, as well as in various regions of the same country. Megaloblastic anemia has been shown to be the most common cause of pancytopenia in studies from northern and southern India, whereas hypersplenism and infections have been implicated to be the most common underlying diseases in a study from western India [5-7]. A similar study on the etiology of pancytopenia from eastern India showed aplastic anemia, followed by megaloblastic anemia, as the most common cause of pancytopenia [8]. Hypoplastic anemia was observed to be the most common cause of pancytopenia in a study conducted in Nepal [9]. The most common cause of pancytopenia was found to be megaloblastic anemia, followed by aplastic anemia, in a study from Pakistan [10]. On the other hand, neoplastic diseases and radiation have been shown to be the most common etiologies of pancytopenia in Europe and Israel [11], and acute myeloid leukemia (AML) was the most common cause of pancytopenia in a Korean study [12]. In a Mexican study, myelodysplastic syndrome (MDS) was the most common cause of pancytopenia, followed by megaloblastic anemia as the second most common cause [13].

Data on the etiology of pancytopenia from various studies in the same geographical region also differ because of the differences in the methodologies employed, diagnostic criteria used, study period, laboratory investigations used, and the department in which the study was conducted. As the severity and underlying cause of pancytopenia determine its treatment and prognosis, documentation of the etiological pattern of pancytopenia plays a crucial role in implementing prompt and appropriate management, unique to pancytopenia patients attending different departments of a healthcare center located in a particular region.

Although a common hematological condition, studies conducted to determine the causes of pancytopenia among patients attending clinical hematology departments in India as well as globally are very scarce. Because pancytopenia patients presenting to clinical hematology departments have different clinical and etiological profiles, they require different diagnostic modalities and therapeutic approaches compared to patients attending other departments.

This study was performed to evaluate the clinico-hematological profile and different etiologies of pancytopenia among adult patients attending a clinical hematology department of a large tertiary care center in Western India.

## Materials and methods

Study design and study population

This prospective, observational study was conducted in the Department of Clinical Hematology at Dr. SN Medical College and associated Mathura Das Mathur Hospital, Jodhpur, Rajasthan, India over a period of four years from April 2018 to March 2022. A total of 546 patients of either sex, aged 18 years or older, and newly detected pancytopenia (hemoglobin level <13.0 g/dL in males, or 12.0 g/dL in females, total leukocyte count (TLC) <4.0 × 10^9^/L, and platelet count <150 × 10^9^/L) attending the inpatient or outpatient departments were included in this study. Previously diagnosed cases of pancytopenia who were under treatment (e.g., drugs, radiation) were excluded from the study.

Study procedure

Ethical approval (SNMC/IEC/IIP/2018/036) for this study was obtained from the Institutional Ethics Committee. Written informed consent was taken from all patients prior to their enrolment in the study. A detailed and relevant medical history was recorded for each participant with special emphasis on symptoms of generalized weakness, shortness of breath, fever, bony pain, and easy bruising and bleeding (e.g., gums, nose, mouth, rectum, vagina). History regarding dietary habit, prior drug intake, exposure to cytotoxic agents and radiation, smoking status, alcohol consumption and other addictions, exposure to recent infections, and blood transfusions was also noted. A detailed physical examination of each participant was performed regarding pallor, icterus, bleeding manifestations (petechiae, purpura, or ecchymoses), lymphadenopathy, sternal tenderness, skin rash, gum hypertrophy, hepatosplenomegaly, and ascites. Complete blood count was recorded for all participants. Anemia was classified as mild if hemoglobin level was >10.0 g/dL to <13.0 g/dL (males) or 12.0 g/dL (females), moderate if hemoglobin level was >7.0 g/dL to 10.0 g/dL, severe if hemoglobin level was >4.0 to ≤7.0 g/dL, and very severe if hemoglobin level was ≤4.0 g/dL. Leukopenia was classified as mild if total leucocyte count (TLC) was >3.0 to <4.0 × 10^9^/L, moderate if TLC was >2.0 to ≤3.0 × 10^9^/L, severe if TLC was >1.0 to ≤2.0 × 10^9^/L, and very severe if TLC was ≤1.0 × 10^9^/L. Thrombocytopenia was classified as mild if platelet count was >100 to <150 × 10^9^/L, moderate if platelet count was >50 to ≤100 × 10^9^/L, severe if platelet count was >10 to ≤50 × 10^9^/L, and very severe if platelet count was ≤10 × 10^9^/L. Other routine blood investigations, including liver function tests, renal function tests, and serum lactate dehydrogenase, and viral markers, including hepatitis B surface antigen, anti-hepatitis C virus antibodies, and anti-human immunodeficiency virus 1 and 2 antibodies, were also performed. Peripheral blood smear (PBS) examination and reticulocyte count were done in each case. Serum iron profile, serum vitamin B12, and folate levels were also measured in each case. Malarial parasite, BM cultures, BM examination including BM aspiration, and trephine biopsy were performed in each case following standard methods. Other relevant and specific investigations such as tropical fever serology, other viral markers, including Epstein-Barr virus and cytomegalovirus, tests for autoimmune diseases, including rheumatoid arthritis factor, antinuclear antibody, and anti-double-stranded deoxyribonucleic acid antibody, tests for hematological malignancies, including flow cytometry, immunohistochemistry, karyotyping and serum protein electrophoresis, chest radiography, and ultrasonography of the abdomen were done wherever indicated.

Statistical analysis

The statistical analysis was performed using the Statistical Package for the Social Sciences (SPSS) software version 21.0 for Windows (SPSS Inc., Chicago, Illinois, USA). Continuous variables were expressed as mean ± standard deviation (SD) or range as appropriate. Categorical variables were indicated as frequency (n) and percentage (%).

## Results

A total of 546 patients with pancytopenia were included in the study group. The following results were recorded and analyzed.

Analysis of age and gender distribution

Out of 546 patients, 305 (56%) were males and 241 (44%) were females (male: female = 1.26:1). The age of the patients ranged from 18 to 93 years, with a mean age of 40.3 years (SD = ±18.5 years). The most common (24%) age group of the patients was 21-30 years, followed by 41-50 years (20%) and 51-60 years (13%) (Table 1).

**Table 1:** Age and gender distribution of the study subjects.

Age groups (years)	Gender		Total (n = 546) n (%)
	Male (n = 305) n (%)	Female (n = 241) n (%)	
≤20	27 (5)	18 (3)	45 (8)
21–30	93 (17)	38 (7)	131 (24)
31–40	55 (10)	43 (8)	98 (18)
41–50	46 (8.5)	63 (11.5)	109 (20)
51–60	27 (5)	44 (8)	71 (13)
61–70	33 (6)	27 (5)	60 (11)
71–80	16 (3)	6 (1)	22 (4)
81–90	6 (1)	2 (0.5)	8 (1.5)
91–100	2 (0.5)	0 (0)	2 (0.5)
Total	305 (56)	241 (44)	546 (100)

Analysis of clinical features

The most common symptom was generalized weakness (68%), followed by fever (30%), shortness of breath (28%), bone pain (20%), and bleeding manifestations (19%). Pallor was the most common clinical finding (79.8%), followed by splenomegaly (22%), bleeding manifestations, including petechiae, purpura, and ecchymoses (15.9%), lymphadenopathy (15.7%), hepatomegaly (12.6%), pedal edema (11.9%), sternal tenderness (10.8%), and icterus (8%) (Table 2).

**Table 2:** Clinical features of patients with pancytopenia at presentation. *Purpura, petechiae, gum bleeding, epistaxis, rectal bleed, and vaginal bleed.

Clinical features	n (%)
Symptoms	
Generalized weakness	371 (68)
Fever	164 (30)
Dyspnoea	153 (28)
Bone pain	109 (20)
Bleeding manifestations*	104 (19)
Weight loss	66 (12.1)
Abdominal pain	28 (5.1)
Mouth ulcers	14 (2.6)
Skin rash	7 (1.3)
Physical findings	
Pallor	436 (79.8)
Icterus	44 (8)
Edema	65 (11.9)
Bleeding/Petechiae/Purpura	87 (15.9)
Lymphadenopathy	86 (15.7)
Hepatomegaly	69 (12.6)
Splenomegaly	120 (22)
Sternal tenderness	59 (10.8)
Ascites	32 (5.9)

Analysis of hematological parameters

Hemoglobin levels ranged from 1.3 g/dL to 12.8 g/dL. Mild-to-moderate anemia (60.1%) was present in a majority of the patients. The remaining 39.9% of patients had severe and very severe anemia (hemoglobin levels ≤7 g/dL) at the time of presentation. TLC ranged from 0.15 × 10^9^/L to 4 × 10^9^/L. Moderate and severe leukopenia was present in a majority of the patients (56%), followed by mild leukopenia in 34.3% of patients and very severe leukopenia in only 9.7% of patients. Absolute neutrophil count (ANC) ranged from 0 to 3.4 × 10^9^/L. Majority (36.3%) of the patients had ANC ranging from >1.0 to ≤1.5 × 10^9^/L followed by >1.5 × 10^9^/L (27.8%), >0.5 to ≤1.0 × 10^9^/L (24.2%), and ≤0.5 × 10^9^/L (11.7%). Platelet count ranged from 1 × 10^9^/L to 143 × 10^9^/L. The majority of the patients (52.6%) had platelet counts ranging from >10 to ≤50 × 10^9^/L, followed by 19.8% with moderate thrombocytopenia. Very severe thrombocytopenia (platelet count ≤10 × 10^9^/L) was present in 18.7% of patients (Table 3).

**Table 3:** Hematological parameters and bone marrow findings of patients with pancytopenia at presentation.

Parameters	n (%)
Hemoglobin (g/dL)	
≤4.0	42 (7.7)
>4.0 to ≤7.0	176 (32.2)
>7.0	328 (60.1)
Total leukocyte count (10^9^/L)	
≤1.0	53 (9.7)
>1.0 to ≤2.0	92 (16.8)
>2.0 to ≤3.0	214 (39.2)
>3.0 to <4.0	187 (34.3)
Absolute neutrophil count (10^9^/L)	
≤0.5	64 (11.7)
>0.5 to ≤1.0	132 (24.2)
>1.0 to ≤1.5	198 (36.3)
>1.5	152 (27.8)
Platelet count (10^9^/L)	
≤10	102 (18.7)
>10 to ≤50	287 (52.6)
>50 to ≤100	108 (19.8)
>100 to <150	49 (8.9)
Peripheral blood smear	
Red blood cell morphology	
Normocytic normochromic	126 (23.1)
Macrocytic	333 (61)
Microcytic hypochromic	54 (9.9)
Dimorphic	33 (6)
White blood cell morphology	
Hypersegmented neutrophils	76 (13.9)
Atypical cells	49 (8.9)
Bone marrow examination findings	
Bone marrow cellularity	
Normocellular	172 (31.5)
Hypercellular	303 (55.5)
Hypocellular	65 (11.9)
Dry tap	6 (1.1)
Other bone marrow findings	
Increased blasts (≥5%)	93 (17)
Dysplasia (≥10%)	22 (4)
Bone marrow fibrosis	60 (11)
Abnormal karyotype	48 (8.8)

The majority of the patients had macrocytic anemia (61%), followed by normocytic normochromic anemia (23.1%) and microcytic hypochromic anemia (9.9%). The remaining patients had dimorphic (showing two red blood cell populations, that is, a combination of microcytic hypochromic and macrocytic normochromic cells) anemia (6%). Vitamin B12 deficiency, MDS, aplastic anemia, and chronic liver disease were among the common causes where macrocytic anemia was noted. Hypersegmented neutrophils were seen in 13.9% of cases, whereas immature cells in peripheral blood were seen in 8.9% of cases. Hypercellular marrow was the predominant marrow finding (55.5%), followed by normocellular marrow (31.5%) and hypocellular marrow (11.9%). Six (1.1%) patients had dry tap marrow. Dysplastic changes in different lineages were seen in 22 (4%) patients. BM fibrosis (MF) grading was done in select patients using reticulin stain on BM biopsy sections. Sixty (11%) patients had marrow fibrosis (MF grade ≥1). Primary myelofibrosis and hairy cell leukemia cases showed the most severe form of MF. Karyotyping was done in select patients using the cytogenetic analysis of BM aspirate samples. An abnormal karyotype was found in 8.8% of patients (Table 3).

Analysis of etiological profile

The most common cause of pancytopenia was acute leukemia in 98 (17.9%) patients. The second most common cause was megaloblastic anemia in 84 (15.4%) patients. Other common causes were aplastic anemia in 60 (11.0%) patients, hypersplenism in 43 (7.8%) patients, multiple myeloma in 36 (6.6%) patients, and MDS in 29 (5.3%) patients. A male preponderance was seen in the cases of acute leukemia, megaloblastic anemia, aplastic anemia, hypersplenism, multiple myeloma, and MDS (Table 4).

**Table 4:** Etiological distribution of patients with pancytopenia according to gender. ICUS: idiopathic cytopenia of undetermined significance; HIV: human immunodeficiency virus

Etiology	Male (n = 305) n (%)	Female (n = 241) n (%)	Total (n = 546) n (%)
Acute leukemia	56 (10.2)	42 (7.7)	98 (17.9)
Megaloblastic anemia	44 (8.1)	40 (7.3)	84 (15.4)
Aplastic anemia	37 (6.8)	23 (4.2)	60 (11.0)
Hypersplenism	27 (4.9)	16 (2.9)	43 (7.8)
Multiple myeloma	21 (3.8)	15 (2.8)	36 (6.6)
Myelodysplastic syndrome	17 (3.1)	12 (2.2)	29 (5.3)
Infections	17 (3.1)	11 (2.0)	28 (5.1)
Non-Hodgkin’s lymphoma	14 (2.6)	10 (1.8)	24 (4.4)
Primary myelofibrosis	12 (2.2)	10 (1.8)	22 (4.0)
Connective tissue disorders	6 (1.1)	14 (2.6)	20 (3.7)
Drug-induced pancytopenia	4 (0.7)	9 (1.7)	13 (2.4)
Metastatic carcinoma	9 (1.6)	3 (0.6)	12 (2.2)
Hemophagocytic lymphohistiocytosis	6 (1.1)	4 (0.7)	10 (1.8)
Paroxysmal nocturnal hemoglobinuria	5 (0.9)	3 (0.6)	8 (1.5)
Hodgkin’s lymphoma	2 (0.4)	6 (1.1)	8 (1.5)
Chronic lymphocytic leukemia	4 (0.7)	3 (0.6)	7 (1.3)
Hairy cell leukemia	5 (0.9)	2 (0.4)	7 (1.3)
Waldenstrom macroglobulinemia	5 (0.9)	1 (0.2)	6 (1.1)
ICUS	2 (0.4)	4 (0.7)	6 (1.1)
Disseminated tuberculosis	2 (0.4)	3 (0.5)	5 (0.9)
HIV infection	4 (0.7)	1 (0.2)	5 (0.9)
Autoimmune marrow fibrosis	1 (0.2)	3 (0.5)	4 (0.7)
Severe iron deficiency anemia	0 (0)	4 (0.7)	4 (0.7)
Histoplasmosis	2 (0.4)	0 (0)	2 (0.4)
Osteopetrosis	1 (0.2)	1 (0.2)	2 (0.4)
Gaucher’s disease	2 (0.4)	0 (0)	2 (0.4)
Sarcoidosis	0 (0)	1 (0.2)	1 (0.2)
Total	305 (56)	241 (44)	546 (100)

## Discussion

This study assessed the clinical profile and etiological spectrum of pancytopenia among adults attending a department of clinical hematology in India. In the study, the mean age of patients was 40.3 years (SD = ±18.5 years). The age range of the patients was from 18 to 93 years, and 70% of patients were below the age of 50 years, with the most common age group (24%) being 21-30 years. The male‑to‑female ratio was 1.26:1 (males, 56%; and females, 44%) with a slight male preponderance. Our results were similar to those reported by Das Makheja et al. where the mean age of patients with pancytopenia was 37.7 years, and the male-to-female ratio was 1.38:1 [2]. In a similar study conducted by Rehmani et al. in Pakistan, the highest percentage of cases of pancytopenia was reported in the 21-30-year age group, with a male-to-female ratio of 1.46:1 [14]. Other studies have also reported similar results [15,16]. In contrast, 41-50 years was the most common age group for cases with pancytopenia in studies by Dasgupta et al. and Tariq et al. [4,17].

The most common symptom of pancytopenia on presentation was generalized weakness (68%), followed by fever (30%), which is in concordance with other similar studies [16,18]. On the contrary, fever was the most common presenting symptom reported in studies by Khodke et al. and Shafiq et al. [15,19]. The next most common symptom was breathlessness (28%), which has also been reported in other previous studies [18,19]. Another common symptom was bleeding manifestations (19%). Niazi and Raziq reported bleeding in 33.7% of pancytopenia patients [16].

Pallor (79.8%) was the most common clinical finding observed in our study, and it was found in almost every case of acute leukemia, megaloblastic anemia, and aplastic anemia. Similar to our observations, pallor was also the predominant clinical finding reported in many previous studies [3,5,15-18,20]. Splenomegaly was the second most common clinical finding found in our study observed in 22% of cases. Jain et al. [20] and other studies [14,17] reported splenomegaly in similar frequency, while Khunger et al. and Khodke et al. observed splenomegaly in 32.5% and 40% of patients, respectively [5,15]. We reported hepatomegaly in 12.6% of cases. Rehmani et al. and Tariq et al. reported similar findings in their studies [14,17]. However, a higher prevalence of hepatomegaly was reported in a few other studies [5,6,15,18].

In the present study, macrocytic anemia was the most common morphologic finding in PBS (61%), followed by normocytic normochromic anemia (23.1%) and microcytic hypochromic anemia (9.9%). Hypersegmented neutrophils were detected in 13.9% of cases and immature cells or blasts in 8.9% of cases in PBS. These findings were consistent with PBS findings reported in other similar studies conducted previously [3,15]. However, hypersegmented neutrophils were detected in 51.3% of cases of megaloblastic anemia in a study by Gayathri and Rao [6].

In our study, BM examination revealed hypercellular marrow (55.5%) as the most common finding, followed by normocellular (31.5%) and hypocellular (11.9%), consistent with the studies by Gayathri and Rao as well as Batool et al., where hypercellularity was also the most frequent BM finding [6,10]. The higher number of cellular marrow smears in this study is likely because of the higher number of cases of acute leukemia and megaloblastic anemia than aplastic anemia. BM biopsy demonstrated MF in 11% of cases. This is in line with the findings of Khodke et al. [15]. An abnormal karyotype was found in 8.8% of patients. However, Safaei et al. in their study showed 31% of patients with abnormal karyotypes [21].

There are varying reports on the underlying etiology and the most common causes of pancytopenia from various parts of the world. A comparison of various causes of pancytopenia observed in different studies is shown in Table 5. In our study, the most common cause of pancytopenia was acute leukemia (17.9%). Acute lymphoblastic leukemia (ALL) was found in 46.9% of patients, whereas 43.8% of cases were AML, 6.1% of cases were acute promyelocytic leukemia (APML), and 3.1% of cases were mixed phenotypic acute leukemia (MPAL) type. Similar to our findings, acute leukemia was the most common etiology (32.2%) of pancytopenia in a study by Khan et al. from Pakistan [22]. In studies by Bae et al. from Korea and Devitt et al. from the United States, AML was the most common cause of pancytopenia found in 25.9% and 26% of cases, respectively [12,23]. Acute leukemia was also found to be the second most common cause of pancytopenia after megaloblastic anemia in Indian studies by Mallik et al. (30.5%) and Chandra et al. (19%) [24,25]. However, in contrast to our findings, other studies from India have observed acute leukemia as a less common etiology of pancytopenia [5,6,15,16,18,20,26]. The higher proportion of acute leukemia in our study may be explained by the fact that this study was conducted in a department of clinical hematology which included a good number of patients referred from other departments, whereas most of the aforementioned Indian studies were conducted either in general medicine departments or pathology departments which cater to different patient populations.

**Table 5:** Comparison of the most common causes of pancytopenia in various studies conducted in India and other countries.

Study	Country	Year	Number of cases	Etiology	
				The most common cause (%)	The second most common cause (%)
Keisu et al. [11]	Israel and Europe	1990	100	Neoplastic diseases and radiation (32%)	Hypoplastic anemia (19%)
Tilak et al. [3]	India	1999	77	Megaloblastic anemia (68%)	Aplastic anemia (7.7%)
Kumar et. al [26]	India	1999	166	Aplastic anemia (29.5%)	Megaloblastic anemia (22.3%)
Khodke et al. [15]	India	2000	50	Megaloblastic anemia (44%)	Aplastic anemia (14%)
Khunger et al. [5]	India	2002	200	Megaloblastic anemia (72%)	Aplastic anemia (14%)
Jha et al. [9]	Nepal	2007	148	Hypoplastic anemia (29%)	Megaloblastic anemia (23.6%)
Tariq et al. [17]	Pakistan	2010	50	Aplastic anemia (36%)	Megaloblastic anemia (16%)
Gayathri et al. [6]	India	2011	104	Megaloblastic anemia (74%)	Aplastic anemia (18.3%)
Jain et al. [7]	India	2013	250	Hypersplenism (29.2%)	Infections (25.6%)
Bae et al. [12]	Korea	2015	1,580	Acute myeloid leukemia (25.9%)	Lymphoma (12.7%)
Rehmani et al. [14]	Pakistan	2015	244	Aplastic anemia (27%)	Megaloblastic anemia (20%)
Dasgupta et al. [8]	India	2015	248	Aplastic anemia (33.5%)	Megaloblastic anemia (21%)
Mallik et al. [24]	India	2016	1,318	Megaloblastic anemia (31.9%)	Sub/aleukemic leukemia (30.5%)
Batool et al. [10]	Pakistan	2018	237	Megaloblastic anemia (27%)	Aplastic anemia (15.6%)
Chandra et al. [25]	India	2019	131	Megaloblastic anemia (25%)	Acute leukemia (19%)
Carretero et al. [13]	Mexico	2019	109	Myelodysplastic syndrome (20.2%)	Megaloblastic anemia (18.3%)
Present study	India	2022	546	Acute leukemia (17.9%)	Megaloblastic anemia (15.4%)

The second most common cause of pancytopenia in our study was megaloblastic anemia (15.4%). Various other studies have shown megaloblastic anemia to be the most common cause of pancytopenia with a reported frequency of 25% to 74% [3,5,6,10,15,17,24,25]. This is probably due to different patient populations in our study attending a clinical hematology department as opposed to general medicine or pathology departments in other studies. In this study, vitamin B12 deficiency was found to be more common than folate deficiency in patients with megaloblastic anemia. This is consistent with the results of similar studies conducted in India and neighboring countries [27]. Nutritional deficiency of vitamin B12 and folate is a well-known cause of megaloblastic anemia leading to pancytopenia. A higher prevalence of nutritional deficiency leading to megaloblastic anemia in the Indian subcontinent reflects low socioeconomic status and an inadequate diet, and might also be attributed to malabsorption syndromes, chronic inflammatory disorders of the intestine, chronic alcoholism, *Helicobacter pylori* colonization, and long‑term use of certain medications that interfere with the metabolism and absorption of vitamin B12 and folate [28].

The third most common cause of pancytopenia in our study was aplastic anemia with a frequency of 11%. A lower prevalence of aplastic anemia (7.7%) was reported in a study by Tilak and Jain [3]. A higher prevalence of aplastic anemia, however, was observed in other previous studies varying from 18.3% to 36% [6,8,9,11,17,26]. Moreover, studies from various parts of the world have reported aplastic anemia as the most common cause of pancytopenia [8,9,14,17,26]. A lower frequency of aplastic anemia in our study may be explained by the fact that the majority of patients (68%) were aged above 40 years as we included adult patients only, whereas the common age of presentation of aplastic anemia is up to 30 years.

In the present study, hypersplenism was found in 7.8% of the cases and was the fourth most common cause of pancytopenia, comparable to the findings of Batool et al., where hypersplenism was found in 5.9% of cases [10]. Dasgupta et al. also reported hypersplenism as the fourth most common cause of pancytopenia but with a higher prevalence (13.7%) than our study [8]. In contrast, Jain et al. reported hypersplenism to be the most common cause of pancytopenia in 29.2% of cases [7]. A lower prevalence of hypersplenism in our study is because of the fact that the most common cause of hypersplenism is chronic liver disease, and patients with this disorder are diagnosed and managed at other departments in our center, such as medicine and gastroenterology departments. Therefore, these patients are not referred to the clinical hematology department for evaluation of pancytopenia, resulting in less contribution of these cases. Additionally, half of our patients (50%) were in the age group of 20-40 years, a younger population, which has a lower prevalence of chronic liver disease, especially due to cirrhosis.

Other common causes of pancytopenia seen in a significant number in this study were hematological malignancies other than acute leukemia such as non-Hodgkin’s lymphoma, MDS, plasma cell myeloma, and myelofibrosis, as well as overwhelming infections.

Strength of the study

Data regarding pancytopenia in the adult population attending a clinical hematology department are limited from India as well as other countries. This study exhibited the clinico‑hematologic profile and various etiologies of pancytopenia in adults patients presenting to the Department of Clinical Hematology at our center, and we hope that our study will be helpful in formulating a diagnostic algorithm and management strategy for patients with pancytopenia at centers where clinical hematology departments do not exist and all cases of pancytopenia are dealt by physicians in general medicine departments. A significant number of cases of pancytopenia will benefit from prompt and appropriate treatment of potentially curable underlying disorders.

Limitations of the study

This study was conducted at a single center with a limited sample size. Therefore, the exact causes of pancytopenia prevalent in the entire region and other parts of India may not have been predicted in the study. Thus, further large and multicenter studies are required to understand the exact causes of pancytopenia among adult patients attending clinical hematology departments.

## Conclusions

Acute leukemia was the most common cause of pancytopenia in our study, followed by megaloblastic anemia and aplastic anemia. The most common age group was 21 to 30 years with a slight male preponderance. Pallor was the most common clinical presentation, followed by generalized weakness, fever, and dyspnea. Macrocytic anemia was the most common morphologic finding on PBS, and most cases had a hypercellular marrow.

This study helped us in understanding the clinical pattern, hematological profile, and various causes of pancytopenia in adults presenting to a hematology department in this region. Large multicenter studies from various regions of the country must be undertaken. Such studies will help in the understanding of the underlying causes of pancytopenia in this setting, thereby better managing patients with critical diseases such as aplastic anemia and hematological malignancies.
